# A Preliminary Analysis of Sociodemographic Characteristics in Patients with Frontotemporal Spectrum Disorder in a population of Buenos Aires, Argentina

**DOI:** 10.1002/alz70857_101701

**Published:** 2025-12-25

**Authors:** Agustin Perez, Gaston Moreno Milicich, Waleska Berrios, Carla Stefani, Diego BAUSO, Mariela Bettini, Marcelo Rugiero, Maria Cecilia Fernandez

**Affiliations:** ^1^ Hospital Italiano de Buenos Aires, Buenos Aires, Argentina; ^2^ Hospital Italiano Buenos Aires, Buenos Aires, Argentina; ^3^ Hospital Italiano de Buenos Aires, Buenos aires, Argentina; ^4^ Hospital Italiano de Buenos Aires, BUenos Aires, Argentina

## Abstract

**Background:**

Frontotemporal spectrum disorder (FTLD) encompasses a group of neurodegenerative conditions affecting the frontal and temporal lobes, leading to distinct clinical syndromes. These include the behavioral variant of frontotemporal dementia (bvFTD), semantic and non‐fluent variants of primary progressive aphasia (svPPA and nfvPPA), frontotemporal dementia with motor neuron disease (FTD‐MND), and tau‐associated disorders such as corticobasal syndrome (CBS) and progressive supranuclear palsy (PSP). Despite advancements in research, sociodemographic data on FTLD remains scarce in our region.

This study evaluated the demographic and clinical characteristics of patients diagnosed with FTLD in a single‐center cohort from Buenos Aires, Argentina.

**Method:**

A retrospective analysis was conducted on 26 patients diagnosed with FTLD at the Neurology Department of our hospital between January 2010 and April 2024. Patients were classified into five subtypes: bvFTD, nfvPPA, svPPA, PSP/CBS, and FTD‐MND. The collected data included demographic profiles, time to diagnosis, family neuropsychiatric history, and pharmacological treatments received.

**Result:**

The most prevalent subtype was bvFTD (42.3%, *n* = 11), followed by nfvPPA (19.2%, *n* = 5), FTD‐MND (19.2%, *n* = 5), PSP/CBS (15.4%, *n* = 4), and svPPA (3.8%, *n* = 1). Women comprised 65.4% of the sample. The mean age at diagnosis was 74.38 years (SD 10.2), with seven patients under 65 years of age (four with bvFTD). The mean education level was 12.8 years (SD 3.89). Fourteen patients exhibited major neurocognitive disorders, and two had positive genetic findings. While 57.7% denied or were unaware of family dementia history, 34.6% were treated with antipsychotics, and 57.7% with antidepressants. The mean time to diagnosis was 2.04 years (SD 1.16).

**Conclusion:**

These findings highlight bvFTD as the most frequent subtype, consistent with prior studies. The shorter diagnostic delay compared to other reports underscores the importance of timely recognition. Expanding the patient registry and evaluating the socioeconomic impact of FTLD are key priorities for future research.

